# Supplementary material for: Oncogenic MYC amplifies mitotic perturbations
Source: Open Biol. 2019 Aug 28;9(8):190136. doi: 10.1098/rsob.190136 (PMC6731591; doi:10.1098/rsob.190136)
Supplement: Supplemental figures [file rsob190136supp1.pdf]

Legends to Supplemental Figures

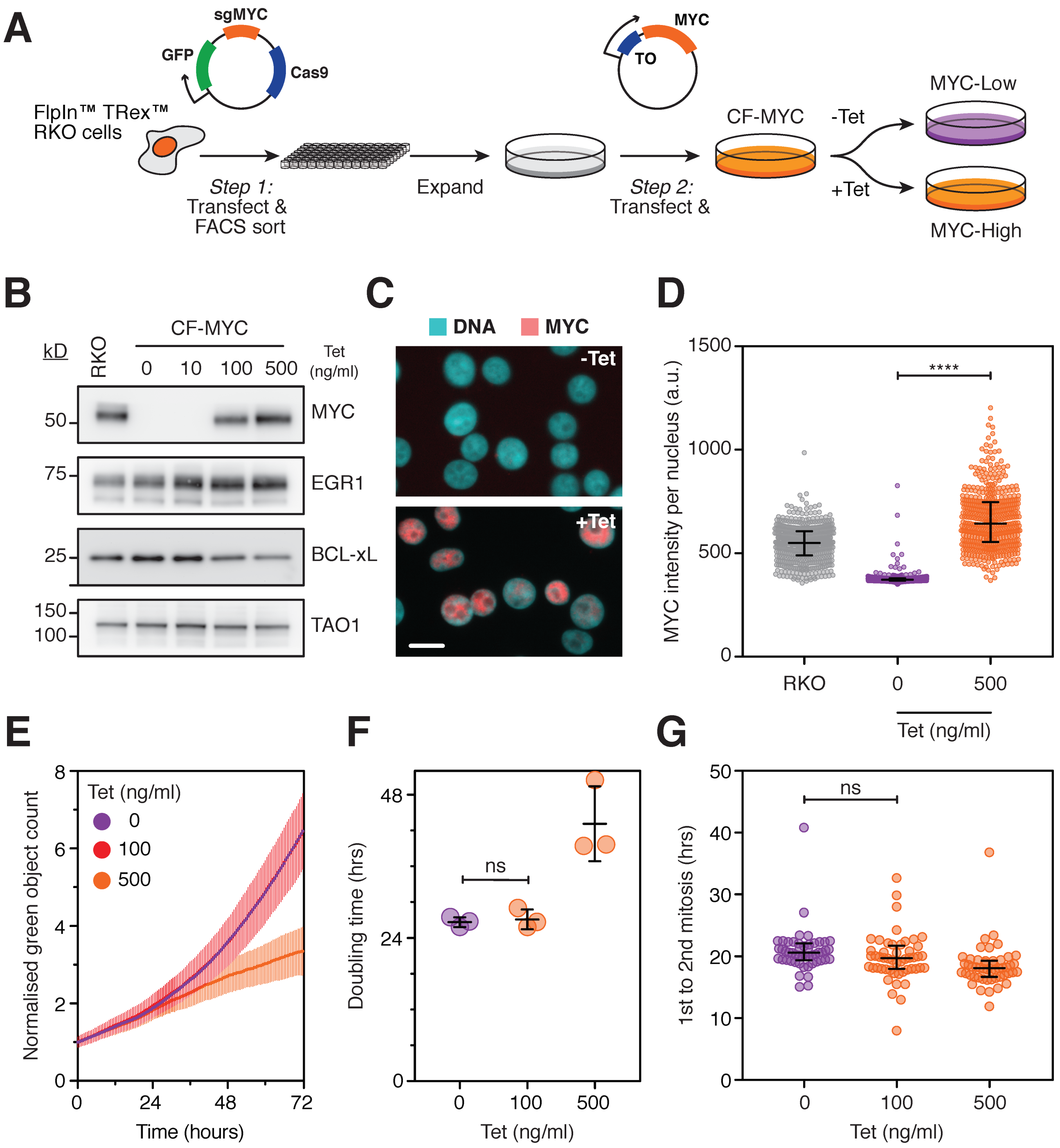

Figure S1. A novel model system to study MYC function. (**A**) Schematic showing the generation of CF-MYC cells whereby both endogenous *MYC* alleles were mutated in FlpIn™ TRex™ RKO cells using CRISPR/Cas-9 gene editing (Step 1), then a tetracycline-inducible MYC transgene was integrated at a pre-inserted FRT site (Step 2). Addition of tetracycline was then used to switch on the transgene thus restoring MYC expression. (**B**) Immunoblots of parental RKO and CF-MYC cells in the presence or absence of tetracycline, analysing expression of MYC and downstream effectors EGR1 and BCL-xL. TAO1 is used as a loading control. (**C**) Immunofluorescence images of CF-MYC cells in the presence or absence of tetracycline. Scale bar 20 µm. (**D**) Scatter dot plot quantitating MYC levels (nuclear immunofluorescence pixel intensities) in RKO and CF-MYC cells in the presence or absence of tetracycline. Symbols show values from individual cells (n=500) while the lines show the median and interquartile ranges. **** p<0.0001, Kruskal-Wallis test with Dunn's multiple comparisons. (**E**) Nuclear proliferation curves of CF-MYC cells expressing a GFP-tagged

histone in the presence or absence of tetracycline. Green object count was determined by time-lapse microscopy, imaging every hour, and the values normalised to the T_0 value, i.e. when imaging started. Values show the mean ± S.D. from three technical replicates. Note that the curves in 0 and 100 ng/ml tetracycline overlap. (**F**) Scatter dot plot showing doubling times of CF-MYC cells in the presence or absence of tetracycline. Values show the mean ± SD from three independent experiments. Note that (E) shows data from one of the experiments used to calculate values in (F). (**G**) Scatter dot plot showing interphase length, as measured by the time interval between the first and second mitoses, in CF-MYC cells in the presence or absence of tetracycline. Values derived from a single experiment representative of two independent replicates, with symbols showing individual cells (n=50) and lines showing the median and interquartile ranges. ns, not significant, Kruskal-Wallis test with Dunn's multiple comparisons.

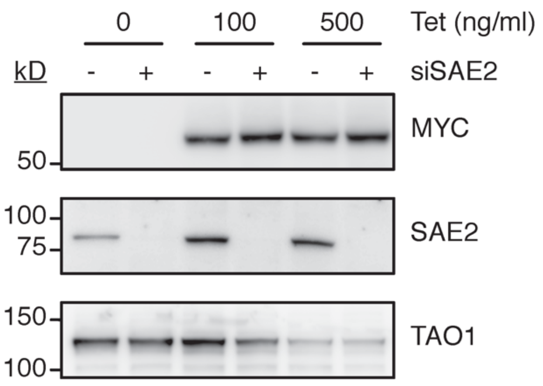

Figure S2. SAE2 suppression combined with MYC overexpression induces cell division failure and cell death. Immunoblots of parental FC-MYC cells in the presence or absence of tetracycline following transfection of siRNAs targeting SAE2. TAO1 is used as a loading control.

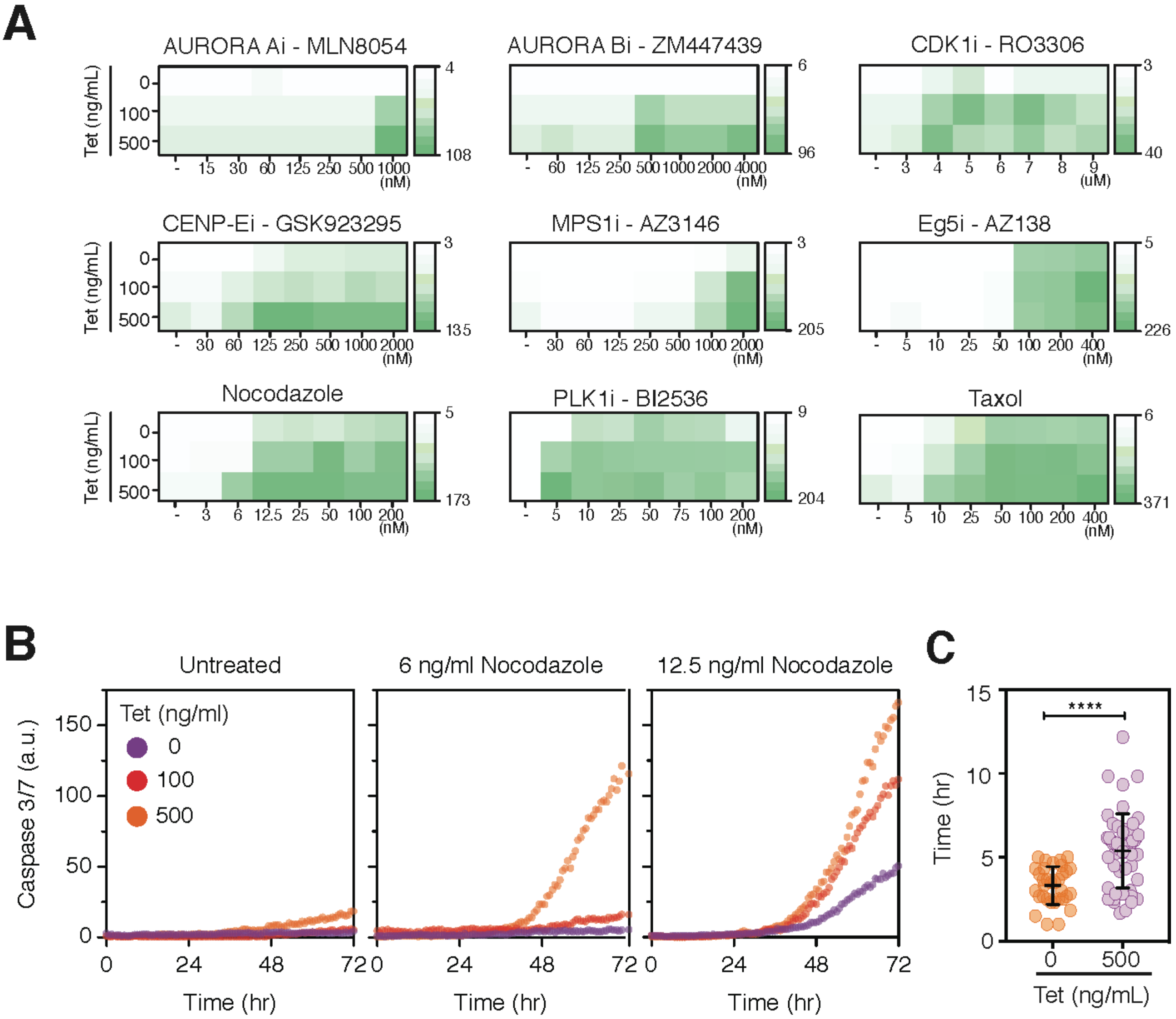

Figure S3. MYC overexpression enhances apoptosis in response to spindle disruption. (**A**) Heat maps derived from concentration matrices whereby FC-MYC cells were exposed to increasing concentrations of anti-mitotic drugs as indicated, in the absence or presence of tetracycline, then analysed as in panel (A). Values represent the fluorescence intensity at 72 hrs. (**B**) Line graphs of FC-MYC cells in the absence or presence of tetracycline, exposed to 0, 6 and 12.5 ng/ml nocodazole then analysed by time lapse imaging in the presence of a fluorescent Caspase 3/7 reporter to quantitate apoptosis. Cells in 96-well plates were exposed to tetracycline for 16 hours prior to the addition of nocodazole and imaging initiated shortly thereafter, capturing four fields of view every hour for 72 hours. Values show the average fluorescence intensities derived from two adjacent wells. (**C**) Scatter dot plot quantitating the duration of mitosis in FC-MYC cells treated with 12.5 ng/ml nocodazole in the absence or presence of 500 ng/ml tetracycline. Only cells that divided then survived were included. Symbols show individual cells (n=33, 0 ng/ml tetracycline; n=50, 500 ng/ml tetracycline), with lines showing the mean ± S.D. **** $p<0.0001$, unpaired t-test with Welch's correction.

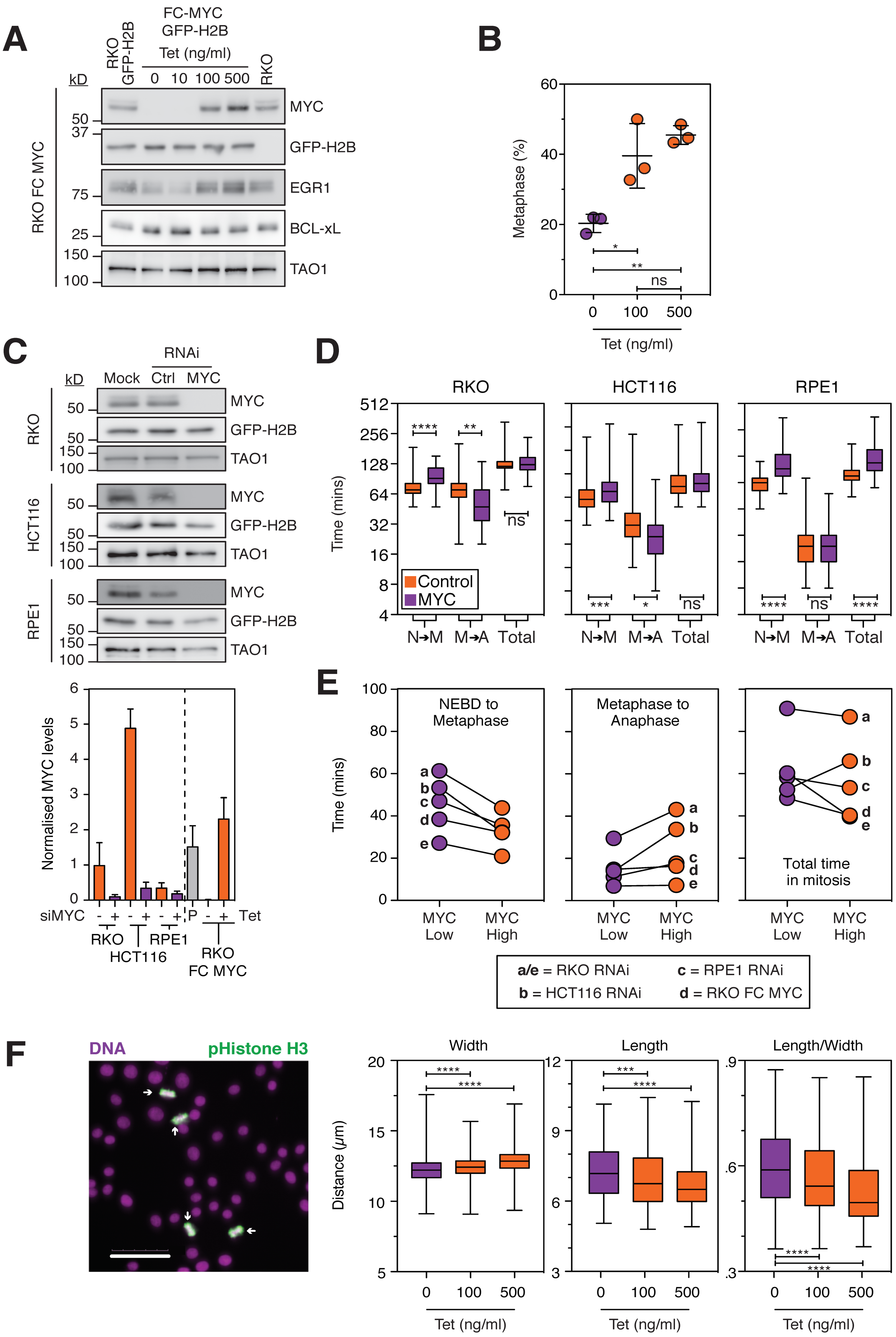
A
RKO GFP-H2B
FC-MYC GFP-H2B
Tet (ng/ml)
0 10 100 500
RKO
kD
RKO FC MYC
MYC
GFP-H2B
EGR1
BCL-xL
TAO1
B
Metaphase (%)
Tet (ng/ml)
ns
C
RNAi
Mock Ctrl MYC
RKO
HCT116
RPE1
Normalised MYC levels
siMYC
Tet
RKO FC MYC
D
RKO
HCT116
RPE1
Time (mins)
Control
MYC
N→M M→A Total
E
NEBD to Metaphase
Metaphase to Anaphase
Total time in mitosis
MYC Low
MYC High
a/e = RKO RNAi
b = HCT116 RNAi
c = RPE1 RNAi
d = RKO FC MYC
F
DNA
pHistone H3
Width
Length
Length/Width
Distance (μm)
Tet (ng/ml)

Figure S4. MYC influences mitotic timing and spindle dynamics. (**A**) Immunoblots of parental RKO, RKO GFP-H2B and FC-MYC GFP-H2B cells in the presence or absence of tetracycline, analysing expression of GFP, MYC and the MYC effectors, EGR1 and BCL-xL. TAO1 is used as a loading control. (**B**) Scatter dot plot quantitating the percentage of the mitotic cells in metaphase in FC-MYC GFP-H2B cells. Each symbol represents an independent experiment, each scoring at least 100 mitotic cells, with lines showing the mean ± S.D. ns, not significant, * $p<0.05$, ** $p<0.01$, Ordinary one-way ANOVA with Tukey's multiple comparisons test. (**C**) Immunoblots and bar graph quantitating MYC levels in RKO, HCT116 and RPE cells, all expressing GFP-H2B, following transfection of siRNAs targeting MYC. TAO1 is used as a loading control. Bar graph shows mean ± S.D. based on two technical replicates from a single experiment, with MYC normalised to TAO1. (**D**) Box-and-whisker plots showing mitotic timings of RKO, HCT116 and RPE1 cells, measuring either the time from nuclear envelope breakdown (NEBD) to metaphase (N→M), metaphase to anaphase (M→A) and the total time in mitosis (Total). Boxes show the median and interquartile ranges while the whiskers show the full range. The data are compiled from three independent experiments measuring an average of 140 cells per condition (range 81-215). ns, not significant, * $p<0.05$, *** $p<0.001$, **** $p<0.0001$, Kruskal-Wallis test with Dunn's multiple comparisons. (**E**) Before-after plots summarizing the mitotic timing data from MYC-Low and MYC-High cells. Data for RKO FC-MYC plus/minus tetracycline cells (d) is derived from Figure 5A. Data for RKO, HCT116 and RPE1 cells plus/minus siRNAs targeting MYC (a, b and c) is derived from (D). Also included is additional data derived from RKO cells plus/minus siRNAs targeting MYC (e) published previously (Topham et al). Symbols show the mean times with the lines connecting the respective populations. (**F**) Exemplar immunofluorescence image used for automated metaphase width and length measurements plus box-and-whisker plots derived from one of the technical replicates used to generate the data in Figure 5C. Image shows FC-MYC cells in the presence of 500 ng/ml tetracycline stained to detect DNA (purple) and phospho-histone H3(Ser10) (green) to identify the mitotic cells (arrows). Scale bar 50 µm. Boxes show the median and interquartile ranges while the whiskers show the full range. *** $p<0.001$, **** $p<0.0001$, Kruskal-Wallis test with Dunn's multiple comparisons.

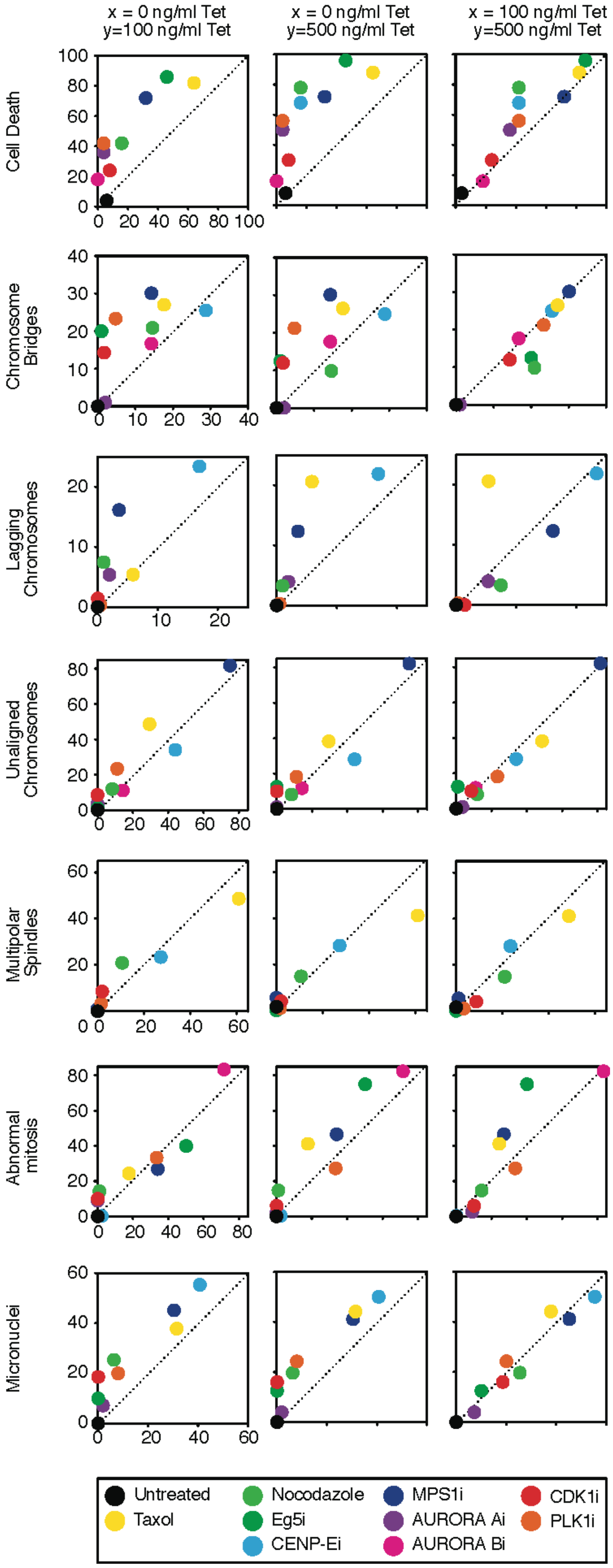

x = 0 ng/ml Tet
y=100 ng/ml Tet
x = 0 ng/ml Tet
y=500 ng/ml Tet
x = 100 ng/ml Tet
y=500 ng/ml Tet
Cell Death
Chromosome Bridges
Lagging Chromosomes
Unaligned Chromosomes
Multipolar Spindles
Abnormal mitosis
Micronuclei
Untreated
Taxol
Nocodazole
Eg5i
CENP-Ei
MPS1i
AURORA Ai
AURORA Bi
CDK1i
PLK1i

Figure S5. MYC overexpression amplifies drug-induced mitotic anomalies. XY plots quantitating the percentage of FC-MYC cells various phenotypes when exposed to the anti-mitotic inhibitors indicated by different coloured symbols. Cell death and chromosome bridge plots for 0 vs. 500 ng/ml tetracycline are reproduced in Figure 6A. Inhibitors used at the following concentrations: Taxol, 10 nM; Nocodazole, 12.5 ng/ml; Eg5i, 100 nM; CENP-Ei, 100 nM; Mps1i 1 µM; Aurora Ai, 1 µM; Aurora Bi, 1 µM; CDK1i, 4 µM; PLK1i, 5 nM. See also Figure S6.

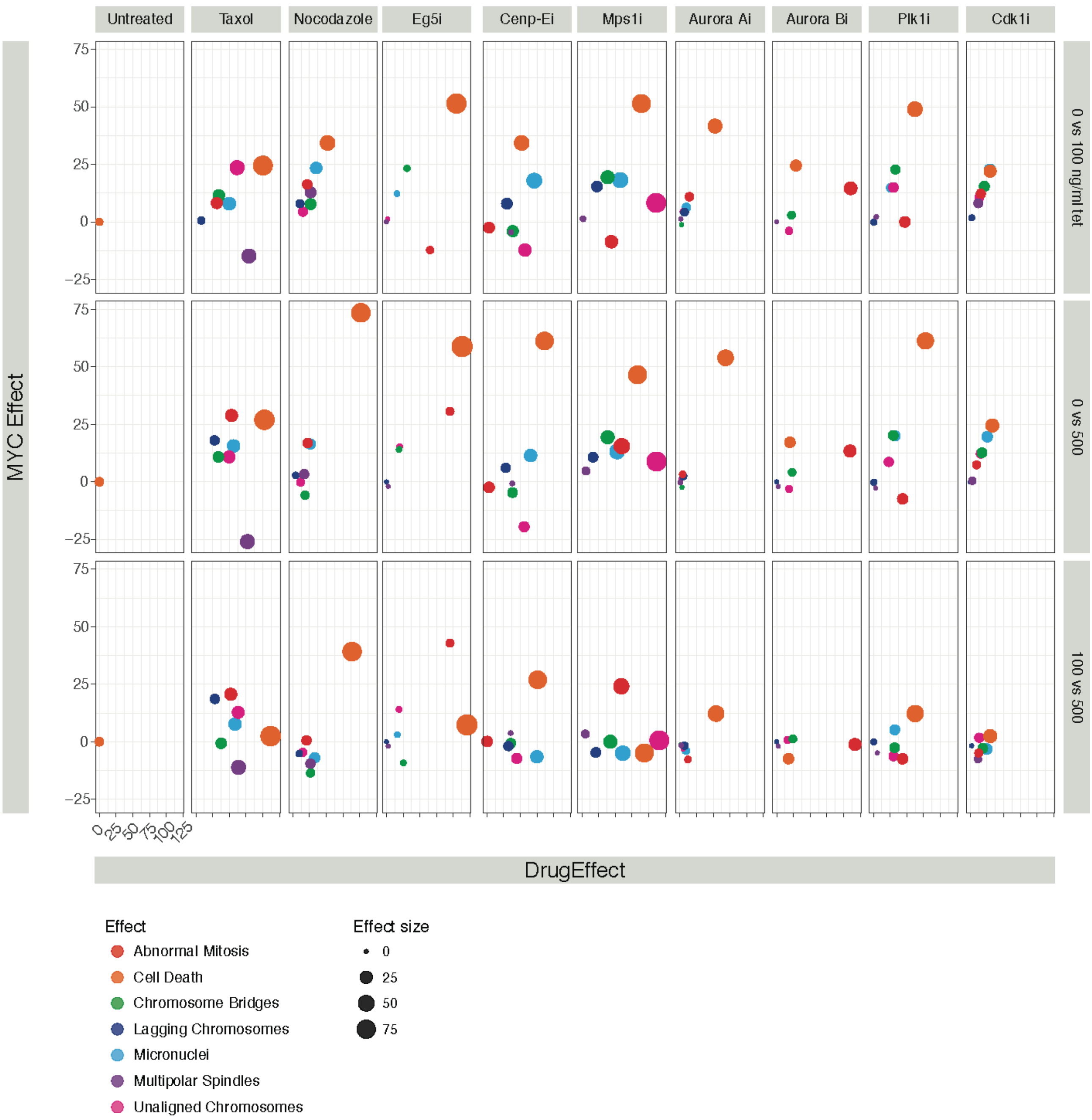

Figure S6. MYC overexpression amplifies drug-induced mitotic anomalies. XY graphs plotting the drug effect against the MYC effect (0 vs. 100, 0 vs. 500 and 100 vs. 500 ng/ml tetracycline) in cells exposed to the drugs indicated with the symbol colours indicating the various phenotypes. The size of the symbol indicates the effect size, i.e. the percentage of the population exhibiting the particular phenotype. Plots analysing 0 vs. 500 ng/ml tetracycline are reproduced in Figure 6C. Inhibitors used at the following concentrations: Taxol, 10 nM; Nocodazole, 12.5 ng/ml; Eg5i, 100 nM; CENP-Ei, 100 nM; Mps1i 1 μM; Aurora Ai, 1 μM; Aurora Bi, 1 μM; CDK1i, 4 μM; PLK1i, 5 nM.

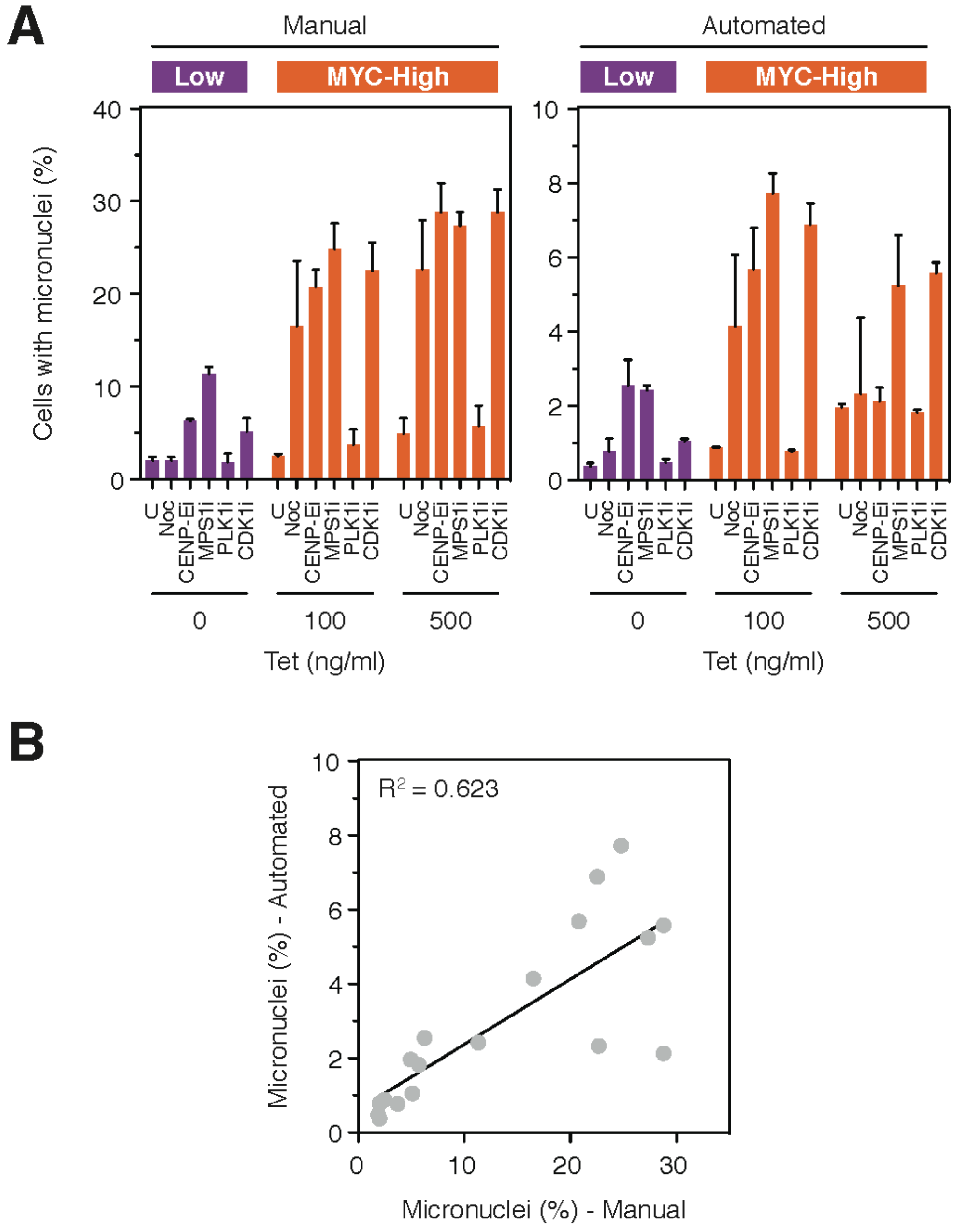

Figure S7. MYC overexpression amplifies micronuclei formation following mitotic perturbations. (**A**) Bar graphs showing manual and automated quantitation of micronuclei in FC-MYC cells, either untreated (U) or exposed to the drugs indicated. Values represent the mean ± S.E.M derived from three technical replicates. (**B**) XY graph plotting the percentage of micronuclei identified by manual vs. automated scoring. R^2 shows the correlation coefficient.

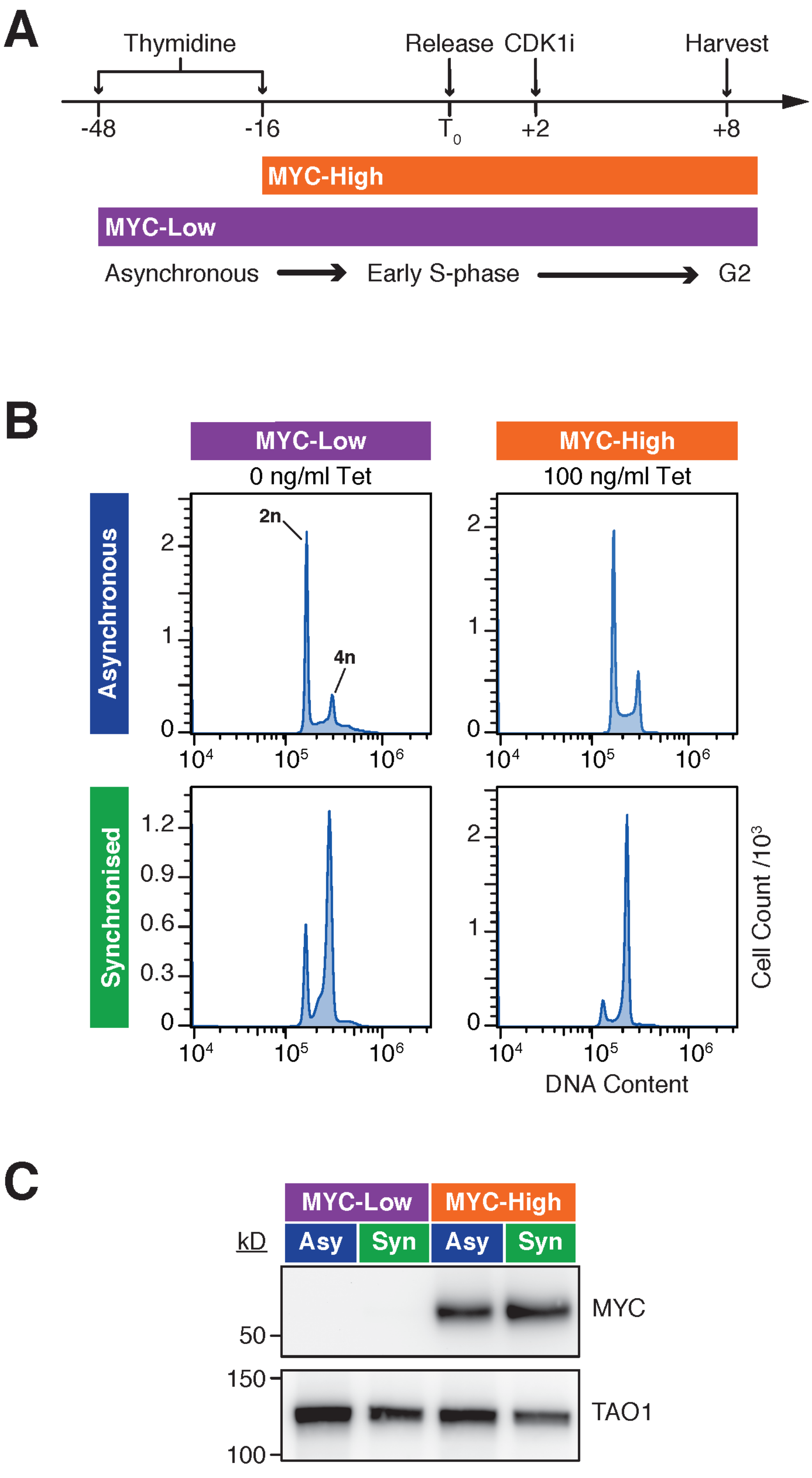

Figure S8. MYC drives mitotic protein networks. (**A**) Time line showing experimental strategy to synchronise FC-MYC cells in G2, either in the absence (MYC-Low) or presence of tetracycline (MYC-High). (**B**) DNA content profiles, as determined by flow cytometry, of FC-MYC cells in the presence or absence of tetracycline indicating the 2n and 4n peaks. Cells were either asynchronous or synchronised in G2 as per the strategy described in panel (A). (**C**) Immunoblots of asynchronous and synchronised FC-MYC showing relative MYC levels. TAO1 was used as a loading control.

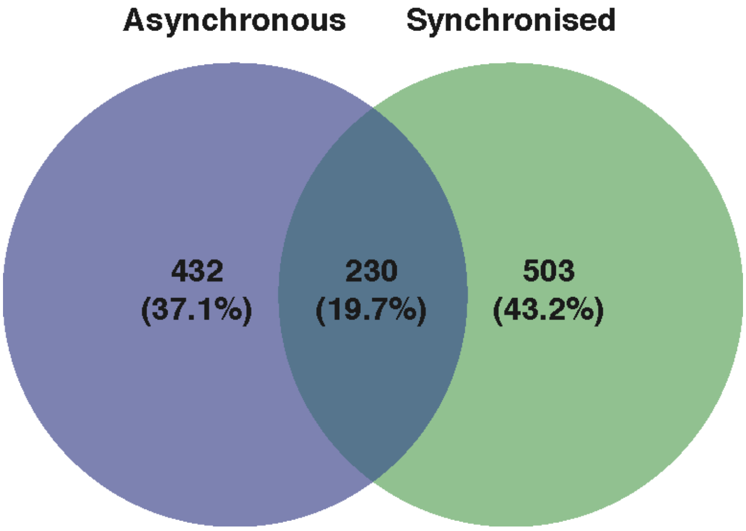

Figure S9. MYC drives mitotic protein networks. Venn diagram comparing asynchronous and synchronised samples and identifying proteins that were differentially expressed or overlapping when comparing MYC-Low with MYC-High cell populations. See also Table S1.

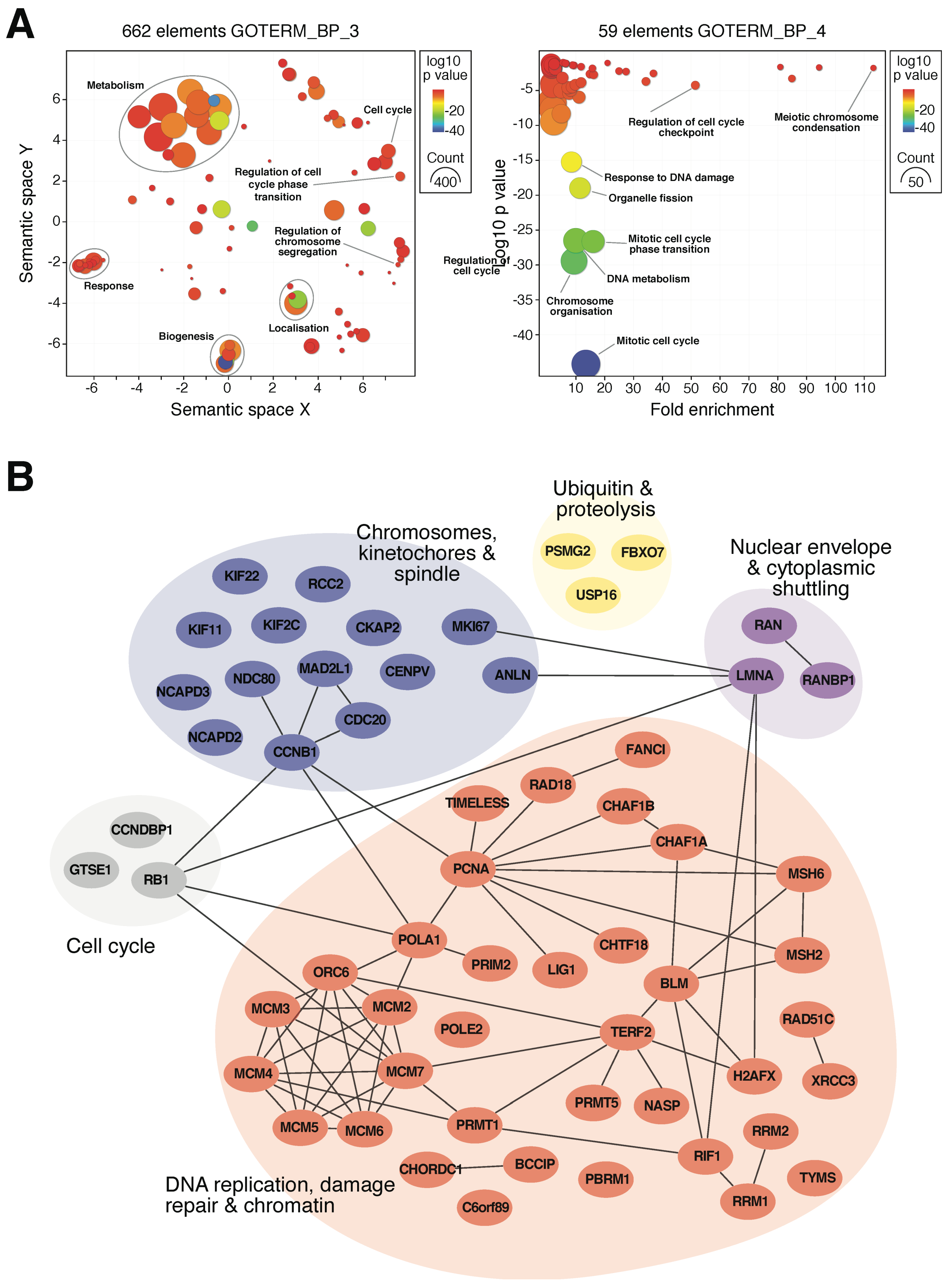

Figure S10. MYC drives mitotic protein networks. (**A**) Ontology analysis of proteins differentially expressed in asynchronous MYC-High and MYC-Low cells. The left panel shows analysis of 662 proteins, highlighting GOTERMS associated with biogenesis, metabolism, protein localisation and cell division. The right panel focuses on 59 proteins associated with cell cycle related GOTERMS. (**B**) Network analysis of 59 proteins associated with cell cycle, highlighting clusters of proteins implicated in DNA replication, chromatin function and DNA damage repair.

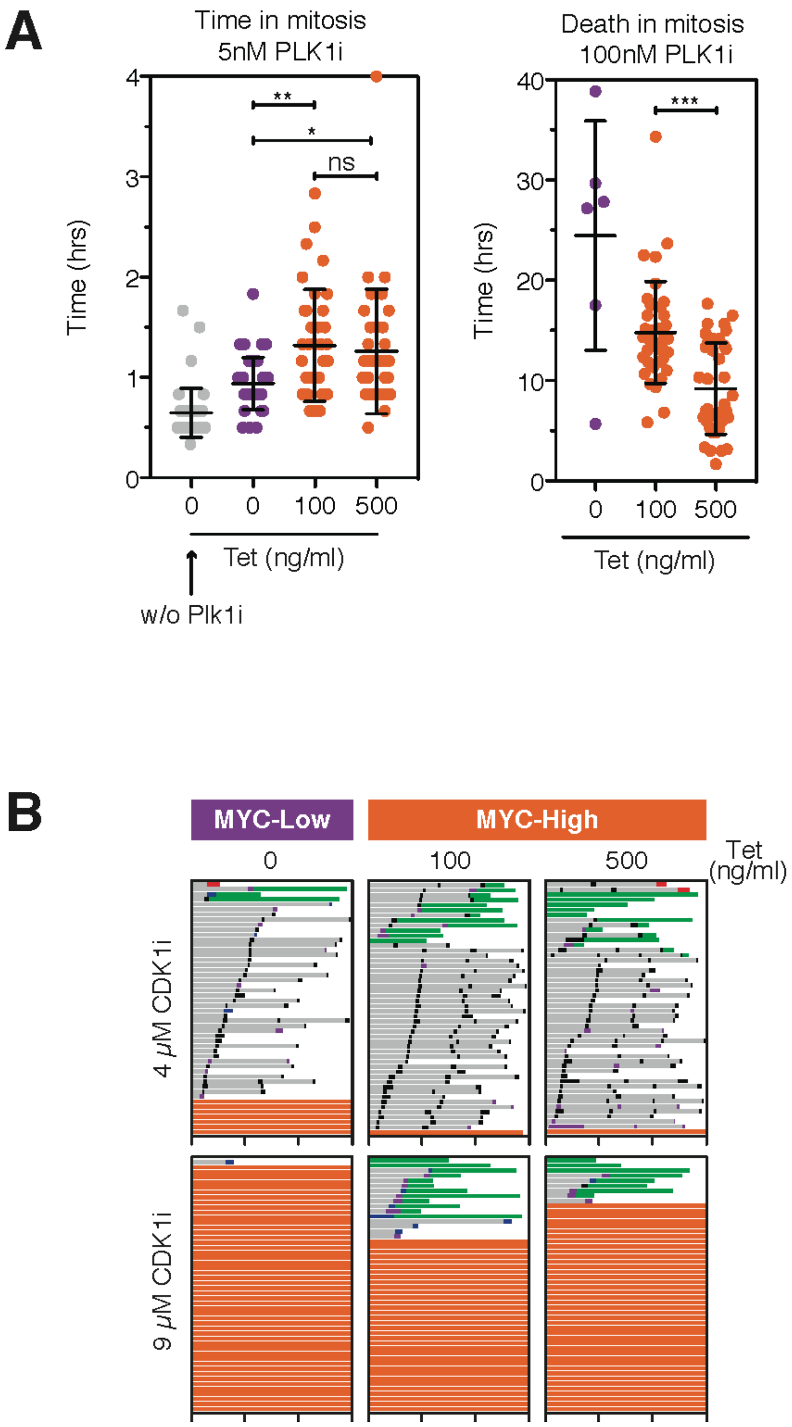

Figure S11. PLK1 inhibition modulates MYC's influence on mitosis. (**A**) Scatter dot plots derived from the data in Figure 9A indicating the time from mitotic entry to either death in mitosis (left graph) or return to interphase (right graph). Each symbol represents a single cell with lines showing the mean ± S.D. ns, not significant, * $p<0.05$, ** $p<0.01$, *** $p<0.001$, Kruskal-Wallis test with Dunn's multiple comparisons. (**B**) Cell fate profiles, as determined by time-lapse microscopy, of FC-MYC cells in the presence or absence of tetracycline, either untreated or following exposure to high and low concentrations of RO3306 (CDK1i). Tetracycline was added for 16 hrs then inhibitors added immediately prior to time-lapse starting at T_0, with images acquired every 10 min. Each horizontal line represents a single cell with the colours indicating cell behaviour. At least 50 cells were analysed per condition.
